# Supplementary material for: Biopsychosocial factors associated with symptom severity in the overlap of non-erosive reflux disease and epigastric pain syndrome: A multicenter cross-sectional study
Source: PLoS One. 2025 Dec 15;20(12):e0338971. doi: 10.1371/journal.pone.0338971 (PMC12704865; doi:10.1371/journal.pone.0338971)
Supplement: Attachment — Submitted filename: Response to Reviewers.docx [file pone.0338971.s003.docx]

**Response to Reviewers**

**Comments from Reviewer 1**

We would like to extend our sincere thanks to you for your time and valuable feedback during the previous round of review. Your constructive comments were instrumental in strengthening our manuscript, and we are truly pleased that you find the revised version satisfactory.

**Comments from Reviewer 2**

**Comment 1:** While the authors have replaced "predictors" in most places, a subtle implication of prediction remains in the Results section (e.g., Page 33/75: "...emerged as statistically significant independent predictors..."). In a cross-sectional study, these are "correlates" or "independent factors associated with," not predictors.

Response: We sincerely thank the reviewer for this meticulous observation. We agree entirely that the language should accurately reflect the associative, non-predictive nature of our cross-sectional findings. As suggested, we have carefully revised the manuscript to replace the term "predictors" with more appropriate terms such as "independent factors associated with" and "correlates" throughout the text, including in the Results section on Page 33. The example sentences now read: "...emerged as statistically significant independent factors associated with..." and we refer to them as "key factors" or "correlates" in subsequent discussions. All these modifications have been highlighted in the revised manuscript for your convenience.

**Comment 2:** The authors have scaled back from prescribing specific treatments... However, the clinical implications could be even more impactful by briefly suggesting how a gastroenterologist might act on a high PHQ-15 score today. A simple statement like, "A high PHQ-15 score should prompt consideration of a multidisciplinary referral to integrated psychiatry or health psychology services, where available, to address the central sensitization component," would provide direct and actionable guidance.

Response: We thank the reviewer for this excellent suggestion to enhance the clinical translatability of our findings. As recommended, we have added the suggested sentence to the 'Discussion' section to provide clear, actionable guidance for clinicians encountering a high PHQ-15 score in practice. The added text is: "In current practice, a high PHQ-15 score should prompt clinicians to consider a multidisciplinary referral to integrated psychiatry or health psychology services, where available, to address the underlying central sensitization and somatic symptom burden." All these modifications have been highlighted in the revised manuscript for your convenience.

**Comment 3:** The results regarding sex are somewhat confusing and could be clarified for the reader. The multivariate model suggests female sex is associated with lower GSRS scores (a "protective effect"), yet the text states, "male patients... tended to have higher GSRS scores than female patients." A sentence acknowledging this contrast and hypothesizing why their cohort might differ (e.g., cultural help-seeking behaviors in men, specific to the overlap phenotype) would resolve this confusion.

Response: We sincerely thank the reviewer for this critical observation. To resolve the confusion completely, we have implemented revisions in both the Results and Discussion sections:

In the Results section, we have replaced the phrase "male patients... tended to have higher GSRS scores" with the more precise and accurate statement: "no statistically significant difference in GSRS scores was observed between male and female patients." This immediately prevents any misinterpretation of a significant trend from the univariate analysis.

In the Discussion section, we have completely revised the corresponding paragraph to provide a coherent interpretation of the nuanced relationship revealed by the multivariate model. The revised text:

1) Explicitly states that the protective association of female sex was identified in the adjusted model but was not apparent in the univariate analysis.
2) Acknowledges and explains this contrast by hypothesizing, as the reviewer suggested, that different help-seeking behaviors (e.g., men in our tertiary care cohort potentially presenting with a higher overall burden) may confound the unadjusted comparison.
3) Provides a biological rationale for the observed protective effect by referencing the potential role of sex-hormone interactions, specifically citing the inhibitory effect of testosterone on the transient receptor potential vanilloid 1 channel (References 43-45).

We believe these combined revisions directly address the reviewer's concern by first ensuring accurate reporting of the result, and then offering a clear and hypothesis-driven explanation for the relationship uncovered by our statistical models.

All these modifications have been highlighted in the revised manuscript for your convenience.

**Comment 4:** The authors' reasoning for keeping two decimal places in percentages is valid from a data transparency standpoint. However, in the main text and abstract, it does impede readability (e.g., "66.67%"). A good compromise would be to report percentages to whole numbers in the abstract and main text for flow, while retaining the precise values in the supplementary data tables.

Response: We thank you for this excellent suggestion to improve the readability of our manuscript. We have fully adopted this compromise. Specifically, all percentages in the Abstract and main Results text that are derived from our own study data and presented in detail within the tables have been rounded to whole numbers to enhance the narrative flow. However, for percentages cited from previous literature (e.g., "as high as 79.4%"), we have retained the original values as reported in the respective sources to preserve accuracy. The changes made in the text have been highlighted for your easy reference. We agree that this approach significantly improves readability without compromising scientific rigor, and we are grateful for your practical advice.
